# 127. Living on the Edge: The Impact of MIC Distributions on Empiric Antibiotic Selection

**DOI:** 10.1093/ofid/ofab466.329

**Published:** 2021-12-04

**Authors:** Karri A Bauer, Levita K Hidayat, Kenneth Klinker, Mary Motyl, C Andrew DeRyke

**Affiliations:** 1 Merck & Co, Inc, Kenilworth, New Jersey; 2 Merck & Co, San Clemente, California; 3 Merck & Co., Inc., Kenilworth, NJ

## Abstract

**Background:**

Due to variability in the precision of an MIC, concern may exist in optimizing PK/PD using standard doses when the MIC is at the susceptibility breakpoint (SBP). This is notable when treating infections in critically ill patients. Evaluating MIC distributions among commonly used antibiotics and accounting for isolates at the SBP represents an additional enhancement to inform empiric therapy. The aim of the study was to evaluate antibiotic susceptibility for commonly used β-lactams against *Pseudomonas aeruginosa* (PA) in a syndromic antibiogram, incorporating MIC distribution.

**Methods:**

20 US institutions submitted yearly up to 250 consecutive targeted Gram-negative pathogens from hospitalized patients as part of the Study for Monitoring Antimicrobial Resistance Trends (SMART) in 2016-2019. MICs were determined by broth microdilution and interpreted using 2021 CLSI breakpoints. The syndromic antibiogram included PA from a blood or respiratory source based on patient location. Based on CLSI guidance, an empiric antibiotic susceptibility threshold of ≥ 90% was deemed optimal.

**Results:**

2,500 PA blood (n=680) and respiratory (n=1,820) isolates were evaluated; piperacillin/tazobactam (P/T), cefepime (FEP), meropenem (MEM), and ceftolozane/tazobactam (C/T) susceptibilities were 69.6%, 74.2%, 75.3%, and 95%, respectively (Figure 1). Isolates with MICs at the SBP were observed in 12.1%, 18.7%, 7.5%, and 6.5% for P/T, FEP, MEM, and C/T, respectively. Susceptibilities were lower when stratified by ICU, 64.8%, 71.2%, 70.7%, and 93.7% for P/T, FEP, MEM, and C/T, respectively with a similar frequency of SBP isolates (Figure 2).

Figure 1. Syndromic antibiogram evaluating *P. aeruginosa* blood and respiratory isolates.

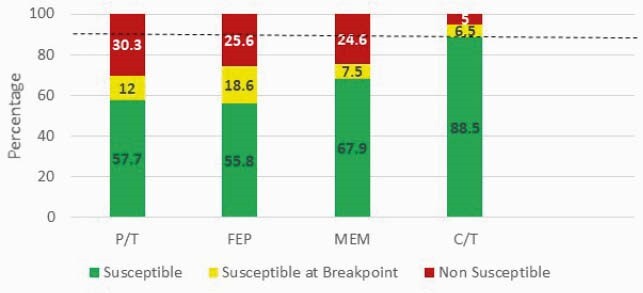

Figure 2. Syndromic antibiogram evaluating *Pseudomonas aeruginosa* blood and respiratory isolates stratified by ICU. *MIC breakpoints used to determine susceptibility included: P/T MIC ≤ 16/4 µg/ml, FEP ≤ 8 µg/ml, MEM ≤ 2 µg/ml, C/T ≤ 4 µg/ml

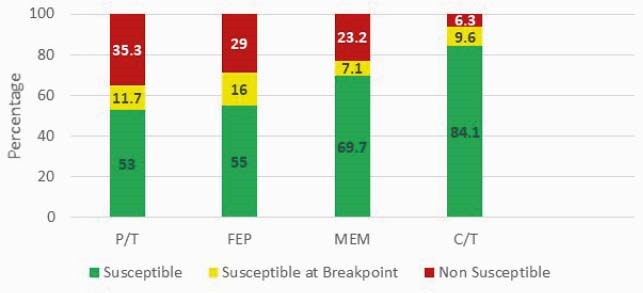

**Conclusion:**

Our analysis demonstrated that first line antipseudomonal agents, P/T and FEP, have susceptibility rates lower than the CLSI recommended threshold. A significant portion of the MICs within the susceptible range are at the SBP. Due to the frequency of baseline resistance and challenge in achieving adequate PK/PD in critically ill patients, clinicians may be concerned with relying on certain antibiotics when the MIC is at the SBP. Antimicrobial stewardship programs should consider incorporating MIC distributions into syndromic antibiograms to better inform empiric therapy recommendations.

**Disclosures:**

**Karri A. Bauer, PharmD**, **Merck & Co., Inc.** (Employee, Shareholder) **Levita K. Hidayat, PharmD BCIDP**, **Merck & Co., Inc.** (Employee, Shareholder) **Kenneth Klinker, PharmD**, **Merck & Co., Inc.** (Employee, Shareholder) **Mary Motyl, PhD**, **Merck & Co., Inc.** (Employee, Shareholder) **C. Andrew DeRyke, PharmD**, **Merck & Co., Inc.** (Employee, Shareholder)

